# Etomoxir-carnitine, a novel pharmaco-metabolite of etomoxir, inhibits phospholipases A_2_ and mitochondrial respiration

**DOI:** 10.1016/j.jlr.2024.100611

**Published:** 2024-07-31

**Authors:** Sung Ho Moon, Xinping Liu, Christopher M. Jenkins, Beverly Gibson Dilthey, Gary J. Patti, Richard W. Gross

**Affiliations:** 1 Division of Bioorganic Chemistry and Molecular Pharmacology, Department of Medicine, Washington University School of Medicine, Saint Louis, MO, USA; 2 Department of Medicine, Washington University School of Medicine, Saint Louis, MO, USA; 3 Department of Chemistry, Washington University, Saint Louis, MO, USA; 4 Siteman Cancer Center, Washington University in St. Louis, Saint Louis, MO, USA; 5 Center for Metabolomics and Isotope Tracing, Washington University in St. Louis, Saint Louis, MO, USA; 6 Department of Developmental Biology, Washington University School of Medicine, Saint Louis, MO, USA; 7 Center for Cardiovascular Research, Washington University School of Medicine, Saint Louis, MO, USA

**Keywords:** carnitine palmitoyltransferase (CPT), etomoxir, off-target effects, etomoxir-carnitine, pharmaco-metabolite

## Abstract

Mitochondrial fatty acid oxidation serves as an essential process for cellular survival, differentiation, proliferation, and energy metabolism. Numerous studies have utilized etomoxir (ETO) for the irreversible inhibition of carnitine palmitoylcarnitine transferase 1 (CPT1), which catalyzes the rate-limiting step for mitochondrial long-chain fatty acid β-oxidation to examine the bioenergetic roles of mitochondrial fatty acid metabolism in many tissues in multiple diverse disease states. Herein, we demonstrate that intact mitochondria robustly metabolize ETO to etomoxir-carnitine (ETO-carnitine) prior to nearly complete ETO-mediated inhibition of CPT1. The novel pharmaco-metabolite, ETO-carnitine, was conclusively identified by accurate mass, fragmentation patterns, and isotopic fine structure. On the basis of these data, ETO-carnitine was successfully differentiated from isobaric structures (e.g., 3-hydroxy-C18:0 carnitine and 3-hydroxy-C18:1 carnitine). Mechanistically, generation of ETO-carnitine from mitochondria required exogenous Mg^2+^, ATP or ADP, CoASH, and L-carnitine, indicating that thioesterification by long-chain acyl-CoA synthetase to form ETO-CoA precedes its conversion to ETO-carnitine by CPT1. CPT1-dependent generation of ETO-carnitine was substantiated by an orthogonal approach using ST1326 (a CPT1 inhibitor), which effectively inhibits mitochondrial ETO-carnitine production. Surprisingly, purified ETO-carnitine potently inhibited calcium-independent PLA_2_γ and PLA_2_β as well as mitochondrial respiration independent of CPT1. Robust production and release of ETO-carnitine from HepG2 cells incubated in the presence of ETO was also demonstrated. Collectively, this study identifies the chemical mechanism for the biosynthesis of a novel pharmaco-metabolite of ETO, ETO-carnitine, that is generated by CPT1 in mitochondria and likely impacts multiple downstream (non-CPT1 related) enzymes and processes in multiple subcellular compartments.

The conversion of nutrients into chemical energy is essential to all life. In higher eukaryotes, the majority of chemical energy is provided by either glucose or fatty acids. Multiple disease processes (e.g., diabetes, obesity, heart failure) have been shown to result from derangements in the physiologic utilization of different mixtures of nutrients, the efficiency of their transformation into chemical energy, and the utilization of this chemical energy by biologically important processes (e.g., muscle contraction, ion gradients, transport processes).

In mammalian cells, the efficiency of ATP and NAD(P)H production and their utilization in diverse metabolic pathways are principal determinants of the bioenergetic status of each cell. Typically, glucose (or other monosaccharides) and fatty acids serve as primary sources of chemical energy. Substantial work has demonstrated the deleterious effects of altering the physiologic ratios of glucose and fatty acid utilization in both a cell-specific and context-dependent manner (1, 2, 3). Most major human metabolic diseases are accompanied by dysfunctional substrate utilization, inefficient conversion of nutrients to chemical energy, and deleterious alterations in redox status and/or membrane potential that collectively compromise bioenergetic efficiency (4, 5).

Various pharmacologic reagents have been routinely utilized to dissect the biochemical mechanisms underlying defects in physiologic functions including cellular energy metabolism. Numerous studies have utilized pharmacologic inhibitors to investigate the significant alterations in substrate utilization and metabolic flux present in obesity, diabetes, and heart disease. Of course, the utility of these pharmacologic reagents is dependent on their specificity which is usually difficult to definitively determine. Etomoxir ((*R*)-2-(6-(4-chlorophenoxy)hexyl)oxirane-2-carboxylic acid) is a widely-employed pharmacologic agent that is typically used to selectively inhibit carnitine palmitoyl transferase 1 (CPT1) and thereby block fatty acid oxidation (FAO) to determine its relative importance in energy production in various disease states. Given that the delivery of long-chain fatty acids into mitochondria is the rate-limiting step for FAO (6, 7), inhibition of CPT1 with etomoxir (ETO) has been extensively used to compare/profile/identify alterations in energy metabolism, cell proliferation, and mitochondrial function due to alterations in fatty acid oxidation. However, multiple published studies including clinical trials have reported off-target effects of ETO independent of its inhibition of CPT1 (8, 9, 10) through mechanisms that are incompletely understood.

In previous work, we have demonstrated important off-target effects of ETO in cellular proliferation and mitochondrial metabolism (11). However, the chemical mechanisms underlying these effects are not well understood. Previously, it has been shown that ETO, which possesses a highly reactive oxirane ring, is subject to nucleophilic attack by CPT1 resulting in subsequent oxirane ring opening and covalent attachment of ETO to the active site serine in CPT1 (12). However, we note that it is likely that the highly reactive oxirane ring of ETO may also be subject to attack by other cellular enzymes (or metabolites) possessing strong nucleophilic residues. Additionally, considering its extensive use in studies throughout the literature, it is surprising that the pharmacologic metabolism of ETO beyond its conversion to ETO-CoA has not, to the best of our knowledge, been significantly investigated.

In the current work, we demonstrate that ETO is predominantly converted to ETO-carnitine through sequential mitochondrial long-chain acyl-CoA synthetase (ACSL) and CPT1 catalytic activities prior to irreversible inhibition of CPT1 by ETO-CoA. Notably, the carnitine adduct of ETO is readily transported into the mitochondria matrix where it nonspecifically targets the functions of various mitochondrial enzymes and respiratory complexes. Collectively, this study demonstrates that ETO-carnitine, a novel pharmacologic metabolite of ETO, contributes to many of the off-target effects of ETO through the generation of novel downstream ETO adducts that can inhibit signaling enzymes and compromise mitochondrial bioenergetics.

## Materials and Methods

### Reagents

(R)-(+)-ETO (sodium salt), oleic acid, and stearic acid were purchased from Cayman Chemical, Co. L-carnitine inner salt, ADP, ATP, fatty acid-free bovine serum albumin (BSA), ammonium acetate, and palmitoyl-1,2,3,4-[^13^C_4_]-L-carnitine were purchased from MilliporeSigma. Lithium and sodium salt hydrates of coenzyme A were purchased from MilliporeSigma and Cayman Chemical, Co, respectively. ST1326, 1-palmitoyl-2-arachidonoyl-*sn*-glycero-3-phosphocholine, 1,3-diolein, and 17:0-lysophosphatidylcholine were obtained from Avanti Polar Lipids, Inc. A bicinchoninic acid protein assay kit and MEM medium were purchased from Thermo Fisher Scientific. Most other reagents and labware were purchased either from Sigma Aldrich or Thermo Fisher Scientific. HepG2 cells were obtained from ATCC. The 63 kDa recombinant human peroxisomal calcium-independent phospholipase A_2_γ (iPLA_2_γ) isoform containing an N-terminal (His)_6_ tag was expressed and purified from Sf9 cells as previously described (13). Recombinant human cytosolic phospholipase A_2_α (cPLA_2_α) containing a C-terminal (His)_6_ tag was expressed and purified from Sf9 cells as previously described (14).

### Expression and purification of recombinant iPLA_2_β(His)_6_ from Sf9 cells

Baculovirus encoding recombinant calcium-independent phospholipase A_2_β (iPLA_2_β) containing a C-terminal (His)_6_ tag was utilized to express iPLA_2_β(His)_6_ in Sf9 cells for purification as previously described (15) with the following modifications. Briefly, Sf9 cells containing recombinant iPLA_2_β(His)_6_ were pelleted by centrifugation (900 × rpm for 10 min), resuspended in lysis buffer (20 mM potassium phosphate, pH 7.8 containing 20% glycerol, 5 mM imidazole, and 2 mM β-mercaptoethanol) with 5 μg/ml leupeptin and 5 μg/ml aprotinin, and sonicated using a Branson sonicator at 30% power (50 × 1 s bursts). The cell sonicate was centrifuged at 100,000*g* for 45 min, the resultant cytosol was diluted 1:1 with lysis buffer containing 500 mM NaCl, and applied to a 3 ml HisPur Ni^2+^-NTA Superflow agarose column equilibrated with lysis buffer containing 250 mM NaCl. After washing the column with 30 ml of lysis buffer containing 5 mM imidazole and 500 mM NaCl, bound protein was eluted in a minimal volume of lysis buffer containing 200 mM imidazole and 500 mM NaCl. The eluate fraction containing iPLA_2_β(His)_6_ was diluted 1:10 with 20 mM imidazole, pH 7.8, containing 20% glycerol, 2 mM DTT, and 1.1 mM CaCl_2_ and applied to a 2.5 ml calmodulin Sepharose column equilibrated with 20 mM imidazole, pH 7.8 containing 20% glycerol, 2 mM CaCl_2_, and 2 mM DTT. After washing with 10 column volumes of 20 mM imidazole, pH 7.8 containing 50 mM NaCl, 20% glycerol, 2 mM DTT, and 1 mM CaCl_2_, bound iPLA_2_β(His)_6_ was eluted with 20 mM imidazole, pH 7.8 containing 200 mM NaCl, 20% glycerol, 2 mM DTT, and 10 mM EGTA. Fractions containing iPLA_2_β(His)_6_ were dialyzed against 20 mM imidazole, pH 7.8, 20% glycerol, 200 mM NaCl, 0.1 mM EDTA, and 2 mM DTT, aliquoted, flash frozen in liquid nitrogen, and stored at −80°C.

### General animal studies

C57BL/6 mice were purchased from Jackson Laboratory. Animal protocols were conducted in strict accordance with the National Institutes of Health guidelines for the humane treatment of animals and were reviewed and approved by the Animal Studies Committee of Washington University.

### Isolation of mitochondria

Heart and liver mitochondria were isolated from C57BL/6 mice (5-8 months-old) by a conventional differential centrifugation method as previously described (16, 17). Briefly, mouse heart or liver tissue was excised from mice euthanized by cervical dislocation in accordance with the NIH guidelines and the Animal Studies Committee at Washington University. Isolated tissues were homogenized in mitochondria isolation buffer (MIB) (10 mM Tris•Cl at pH 7.4 with 0.21 M mannitol, 0.07 M sucrose, 0.1 mM potassium-EDTA, 1 mM EGTA, and 0.5% fatty acid-free BSA) via 10 strokes of a Teflon homogenizer using a rotational speed of 120 rpm. The homogenates were centrifuged at 850 *g* for 7 min and the supernatant was collected and centrifuged at 10,000 *g* for 10 min. The resultant mitochondrial pellet was resuspended in MIB buffer without fatty acid-free BSA. For liver mitochondria, the reconstituted mitochondria in MIB buffer were centrifuged again at 7,500 *g* for 10 min and the mitochondrial pellet was reconstituted in MIB buffer without fatty acid-free BSA. The quantity of mitochondrial protein was determined by a bicinchoninic acid protein assay method using bovine serum albumin as a standard.

### Fatty acylcarnitine and ETO-carnitine synthesis assay

Isolated mouse heart or liver mitochondria (120 μg protein/ml) were placed in mitochondria assay buffer containing 10 mM HEPES (pH 7.2), 0.23 M mannitol, 0.07 M sucrose, 0.5 mM ATP, 1.25 mM ADP, 5 mM MgCl_2_, 2 μM rotenone, 0.5 mM L-carnitine, 0.5 mM reduced coenzyme A (CoASH), and 5 mM succinate with or without ETO (0.1% DMSO vehicle) and incubated for the indicated times at 35°C. Acylcarnitine production was terminated by adding MeOH (50% v/v final) followed by the addition of an internal standard for acylcarnitine, palmitoyl-1,2,3,4-[^13^C_4_]-L-carnitine. After vigorous vortexing, insoluble mitochondrial debris was pelleted by centrifugation at 4000 *g* for 10 min. The supernatant was collected and diluted by the addition of water (20% MeOH final). Lipids present in the supernatant were isolated using a C18 SPE column (Sep-Pak® C18 Cartridges from Waters) prior to measurement of fatty acylcarnitines and ETO-carnitine by precursor ion scanning at *m/z* 85.0 using ESI-MS spectrometry in the positive ion mode (triple stage quadrupole [TSQ] Quantum Ultra Mass Spectrometer from Thermo Fisher Scientific). The quantities of fatty acylcarnitines were determined by comparing their relative intensities to palmitoyl-1,2,3,4-[^13^C_4_]-L-carnitine internal standard at MS^1^.

### Preparative synthesis and purification of ETO-carnitine

Intact mouse liver or heart mitochondria were isolated using the differential centrifugation method described above. Pelleted mitochondria were then resuspended (200–300 μg protein/ml) in mitochondria assay buffer containing 10 mM Hepes (pH 7.4), 0.23 M mannitol, 0.07 M sucrose, 0.25 mM reduced coenzyme A (CoASH), 0.5 mM L-carnitine, 2 μM rotenone, 5 mM succinate, 1.25 mM ADP, 0.5 mM ATP, 5 mM MgCl_2_, and 20 μM (for heart mitochondria) or 50 μM (for liver mitochondria) ETO prior to incubation at 37°C for 30 min. In some cases, frozen mitochondria were utilized for the generation of ETO-carnitine. The CPT1 reaction was terminated by the addition of methanol (final concentration 80%, v/v), vigorously vortexed and insoluble debris was pelleted by centrifugation at 4,000*g* for 10 min. The supernatant was then collected, diluted with water (to yield 20% methanol (v/v) final concentration), and loaded onto a C18-SPE column (Sep-Pak® C18 Cartridges from Waters). Lipid components containing acylcarnitines were eluted with 90% methanol, dried under N_2_ stream, and re-extracted by a Bligh-Dyer method (3X) (methanol/water/chloroform, 1:1:1 (v/v/v)). The chloroform layer was collected and dried under a nitrogen stream. ETO-carnitine in the extracts was resolved by C18 reversed HPLC (Kinetex ® EVO C18 LC column (2.6 μm particles, 250 × 4.6 mm) from Phenomenex) using a linear gradient of solvent A (H_2_O), solvent B (acetonitrile: methanol: H_2_O, 2:1:1 (v/v/v), 5 mM ammonium acetate) at 0.8 ml/min flow rate. The linear gradients of the mobile phase for the resolution of ETO-carnitine are as follows: 0 min, 32% A and 68% B; 20 min, 26% A and 74% B; 25 min, 100% B. The quantitation of ETO-carnitine was performed by TSQ ESI-MS (MS^1^) analysis utilizing palmitoyl-1,2,3,4-[^13^C_4_]-L-carnitine as an internal standard.

### C18 reversed-phase LC-MS^n^ mass spectrometry

LC-MS analyses were performed using an LTQ-Orbitrap mass spectrometer (Thermo Fisher Scientific, San Jose, CA) equipped with an ACQUITY UPLC system (Waters, Milford, MA). Briefly, chromatographic separations were performed on a C18 reversed-phase column (Ascentis Express, 2.7 μm particles, 150 × 2.1 mm) using a linear gradient of solvent A (0.1% glacial acetic acid in water) and solvent B (0.1% glacial acetic acid in acetonitrile) at a flow rate of 0.2 ml/min with the following solvent gradient: 0.0–1.0 min, 5%–22% B; 1.0–7.0 min, 22%–26% B; 7.0–7.1 min, 26%–40% B; 7.1–20.0 min, 40%–60% B; 20.0–21.0 min, 100% B; 21.0–34.0 min, 100% B; 34.0–35.0 min, 100% B to 5% B followed by 15 min isocratic re-equilibration at 5% B. The sample injection volume was 10 μl and the autosampler tray temperature was maintained at 4°C throughout the analysis. The LTQ ion source was operated in the positive ion mode at sheath, auxiliary, and sweep gas flow values (arbitrary units) of 35, 5, and 1, respectively. The capillary temperature was set to 250°C, and the electrospray voltage was 4.1 kV. Capillary voltage and tube lens voltage were set at 30 V and 110 V, respectively. The mass spectrometer was calibrated using the manufacturer’s recommended positive mode calibration solution containing L-methionyl-arginyl-phenylalanyl-alanine acetate, Ultramark 1,621, and caffeine. The mass accuracy was within 5 ppm at mass values from *m/z* 130 to 2000. Resolving powers of 30,000 in full scan mode and 15,000 in MS^n^ (n = 2–3) mode were used. For MS^n^ (n = 2–3) analyses, a normalized collision energy of 30% was applied and the activation time was set at 30 ms with an activation parameter of q = 0.25. Data acquisition was performed using an Xcalibur operating system, v2.1 (Thermo Fisher Scientific).

### Phospholipase A_2_ activity assay

Recombinant iPLA_2_γ (0.5 μg), iPLA_2_β (0.64 μg), or cPLA_2_α (0.68 μg) were diluted in reaction buffer containing 10 mM Hepes (pH 7.4), 10% glycerol, 50 mM KCl, and 1 mM DTT. For the cPLA_2_α activity assay, 0.5 mM CaCl_2_ was added to the reaction buffer without DTT. These enzymes were pre-incubated with various concentrations of either ETO, ETO-carnitine, or DMSO vehicle alone for 10 min. PLA_2_ reactions were initiated by the addition of SUV substrate (50 μM 1-palmitoyl-2-arachidonoyl-*sn*-glycero-3-phosphocholine/12 μM 1,3-diolein) followed by incubation at 37°C while shaking (120 rpm) for 10 min. Reactions were terminated by the addition of methanol/chloroform (1:1, v/v) followed by addition of a lysophosphatidylcholine (LPC) internal standard (17:0 LPC). LPCs from the reaction were extracted by a Bligh-Dyer method and quantitated by TSQ ESI-MS spectrometry (17).

### Acid hydrolysis of the ETO-carnitine oxirane ring

Hydrolytic opening of the oxirane ring of ETO-carnitine was performed as previously described for ETO (18). HPLC-purified ETO-carnitine (100 pmol) was placed in 400 μl 0.2 M H_2_SO_4_ and heated at 60 °C for 2 h with gentle magnetic stirring. The resultant products were dried under a nitrogen stream, reconstituted in chloroform/methanol (50:50), and analyzed by ESI-MS spectrometry (TSQ Quantum Ultra mass spectrometer). For LC-MS^n^ analysis, the reaction products were separated utilizing a C18 reversed-phase column prior to mass spectrometric analysis using an LTQ-Orbitrap as described above.

### Mitochondrial high resolution respirometry

Mitochondrial high resolution respirometry was performed utilizing an OROBOROS® Oxygraph 2K respirometer (Innsbruck, Austria) as described previously (19). Isolated heart mitochondria (50 μg protein for palmitoylcarnitine substrate or 120 μg protein for palmitoyl-CoA substrate) were resuspended in mitochondrial respiration buffer (MiR05: 110 mM sucrose, 60 mM potassium lactobionate, 20 mM taurine, 20 mM Hepes, 10 mM KH_2_PO_4_, 3 mM MgCl_2_, 0.5 mM EGTA, 1% fatty acid-free BSA, pH 7.1) in each of the 2-ml chambers of the respirometer in either the absence (DMSO vehicle) or presence of 0.5 μM ETO-carnitine. The oxygen concentration in the chambers and the O_2_ consumption rate at state 2 and ADP-activated state 3 respiration were monitored with sequential addition of substrates (15 μM palmitoylcarnitine/5 mM malate or 15 μM palmitoyl-CoA/5 mM malate/0.5 mM L-carnitine) and 1.25 mM ADP at the times indicated in the figures. The oxygen consumption rate was calculated as a time derivative of the oxygen concentration using DatLab 6.0 Analysis software (OROBOROS®, Innsbruck, Austria).

### Cellular production of ETO-carnitine

HepG2 cells were plated on 10 cm dishes and cultured in minimum essential medium containing 10% heat-inactivated FBS and penicillin-streptomycin (1X). Cells at 50%–60% confluency were incubated in fresh culture media containing 0.25 mM L-carnitine and various concentrations (5, 25, or 200 μM) of ETO or vehicle alone (DMSO, 0.1% v/v) for 24 h. Culture media and cells were collected separately and the lipid contents were repeatedly extracted by utilizing a Bligh-Dyer method (4X) in the presence of an internal standard, palmitoyl-1,2,3,4-[^13^C_4_]-L-carnitine and then purified using a C18-SPE column. The quantity of ETO-carnitine produced was measured by LC-MS analysis as described above. Accumulation of intracellular ETO was determined by LC-MS in the negative-ion mode utilizing an external ETO standard.

### Statistical analyses

Values are expressed as mean ± S.E.M. The significance of experimental observations was determined by a Student's *t* test, and results were considered significant at *P* < 0.05.

## Results

### Mitochondrial-mediated generation of ETO-carnitine from ETO

**Fig. 1:**
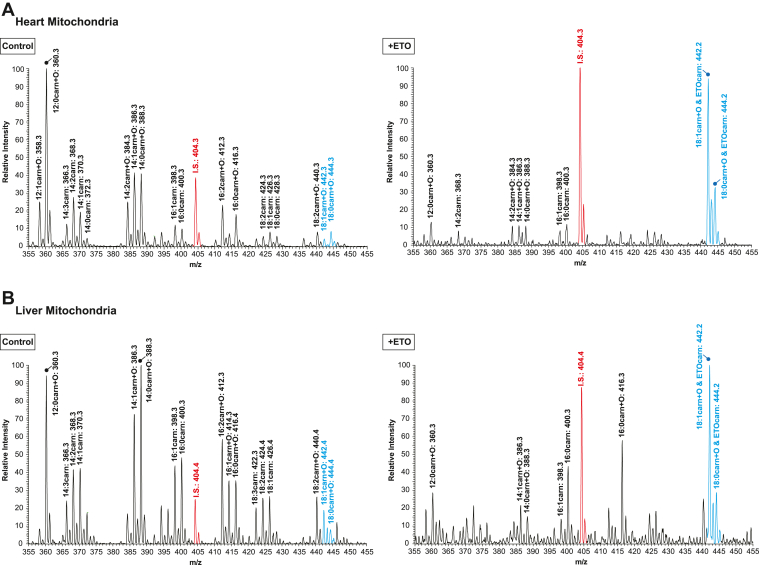
Alterations in mitochondrial acylcarnitine content and the generation of etomoxir-carnitine in the presence of etomoxir. Mitochondria from mouse heart and liver were isolated by differential centrifugation as described in “Materials and Methods”. Intact heart (A) and liver (B) mitochondria were incubated in the absence (control, left panels) or presence of either 40 μM or 60 μM etomoxir (right panels), respectively, for 30 min. Mitochondrial production of acylcarnitines was terminated by adding methanol (50% v/v final concentration) and the lipids present in the resultant methanol solution were collected after removal of precipitated protein by centrifugation. Fatty acylcarnitines in the supernatant were isolated by an SPE column and analyzed by ESI mass spectrometry. Representative mass spectra for fatty acylcarnitines and the putative etomoxir-carnitine (ETOcarn) are identified as molecular species and their corresponding protonated molecular masses (*m/z*), that is, [M+H]^+^. Relevant fatty acylcarnitines and putative ETOcarn molecular species are highlighted in blue. Palmitoyl-1,2,3,4-[^13^C_4_]-L-carnitine (*m/z* 404.3) was used as an internal standard (I.S. in red).

Intact mitochondria were isolated from mouse heart (CPT1B-abundant (20)) and liver (CPT1A-abundant (21)) by conventional two-step differential centrifugation methods as described in “Materials and Methods”. Mitochondrial fatty acylcarnitine levels in the absence (Control) or presence of ETO, an irreversible selective inhibitor of CPT1, were monitored with a triple stage quadrupole mass spectrometer by precursor ion scanning at *m/z* 85.0 for 5-hydroxy-2*H*-furan-1-ium, [C_4_H_5_O_2_]^+^ which is diagnostic for acylcarnitines as previously described (22). In the absence of ETO, mitochondria accumulated a variety of fatty acylcarnitines which included nonhydroxy and 3-hydroxy fatty acylcarnitines generated from FAO of endogenous fatty acids. Following incubation with ETO, the overwhelming majority of the observed fatty acylcarnitines dramatically decreased due to ETO-mediated inhibition of CPT1, which catalyzes the rate-limiting step of mitochondrial FAO. Remarkably, we observed the presence of two predominant peaks at *m/*z 442.2 and 444.2 which were significantly increased in the presence of ETO (Fig. 1) and contrasted with the broad-based decrease in fatty acylcarnitines. Given that ETO contains a carboxylic acid group and is known to generate ETO-CoA by Mg^2+^/ATP-activated ACSL in the presence of reduced coenzyme A (CoASH) (23), we hypothesized that ETO-CoA might be a substrate for active CPT1. Therefore, we speculated that ETO-CoA was further metabolized to ETO-carnitine by sequential catalysis by acyl transfer of the etomoxiryl group (of ETO-CoA) to L-carnitine by CPT1.

### Identification of ETO-carnitine by high performance LC-MS^n^ with HRAM analyses

**Fig. 2:**
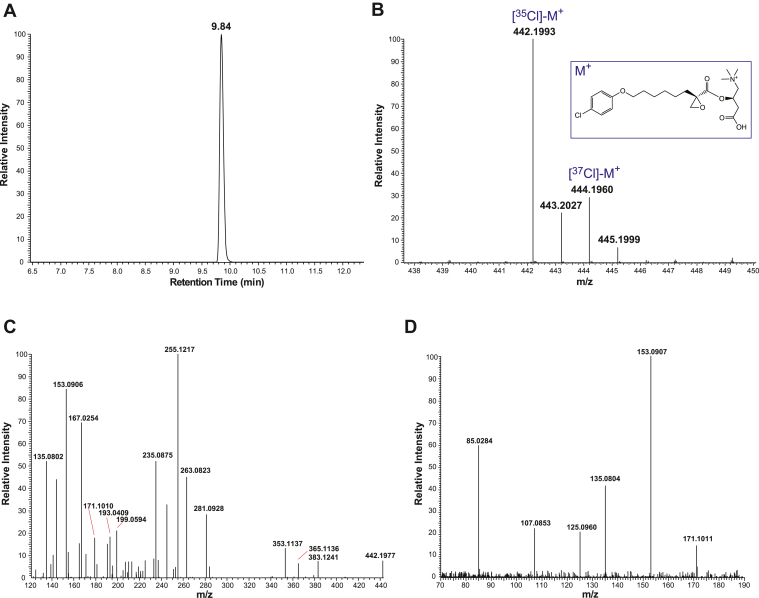
Identification of etomoxir-carnitine by LC-HRAM (high-resolution accurate mass) mass spectrometry. Etomoxir-carnitine was generated utilizing isolated heart mitochondria as described in “Materials and Methods”, extracted by SPE and purified by HPLC by monitoring absorbances at 204 nm and 234 nm wavelengths. The collected etomoxir-carnitine was further analyzed for its elution time by UHPLC followed by accurate mass analysis by HRAM mass spectrometry. A: LC retention time of ETO-carnitine was determined utilizing LC-MS. The extracted ion chromatogram for ETO-carnitine (*m/z* 442.1991 with a 5 ppm mass window) is shown. B: HRAM MS^1^ spectrum of ETO-carnitine. Note that etomoxir-carnitine exists as two major stable isotopes of chlorine (i.e., ^35^Cl and ^37^Cl). C: HRAM MS^2^ spectrum of [^35^Cl]-M^+^ (*m/z* 442.1993 in the MS^1^ spectrum (B)). D: HRAM MS^3^ spectrum of the ion at *m/z* 255.1217 in the MS^2^ spectrum (C). Predominant fragment ions and their pathways of formation are described in the “Supplemental Data.”

Considering that the predicted molecular masses of the two Cl isotopes (^35^Cl and ^37^Cl) of ETO-carnitine ((*R*)-3-carboxy-2-(((*R*)-2-(6-(4-chlorophenoxy)hexyl)oxirane-2-carbonyl)oxy)-*N*,N,*N*-trimethylpropan-1-aminium, C_22_H_33_ClNO_6_^+^ with *m/z* 442.2 for the ^35^Cl-containing isotope and *m/z* 444.2 for the ^37^Cl-containing isotope) are virtually identical to those of the naturally occurring fatty acylcarnitines, hydroxy-C18:1 carnitine and hydroxy-C18:0 carnitine by mass spectrometry with moderate mass resolution (Fig. 1), we sought to unambiguously discriminate these two different metabolites using a high-resolution mass spectrometry. Accordingly, we determined the retention times and accurate masses of the putative ETO-carnitine peaks by C18 reversed-phase UHPLC and high-resolution accurate mass (HRAM) mass spectrometry (Fig. 2). Briefly, isolated mouse heart mitochondria were incubated with 40 μM ETO, the reactions terminated by the addition of methanol, and the lipid contents collected by solid phase extraction prior to purification by UHPLC and HRAM analysis. The two putative Cl isotopes of ETO-carnitine co-eluted (as expected) at 9.8 min (Fig. 2A) and their accurate masses (*m/z*) were determined. Moieties with *m/z* 442.1993 (Δm/m = 0.45 ppm) and *m/z* 444.1960 (Δm/m = 0.23 ppm) were identified (Fig. 2B) which corresponded favorably with their theoretical masses of *m/z* 442.1991 and *m/z* 444.1961, respectively. Next, we employed MS^n^ tandem mass spectrometric analyses to further characterize the chemical structure of ETO-carnitine. Signature fragment ions from MS^2^ and their proposed fragments are shown in Fig. 2C and supplemental Fig. S1 including the following peak: *m/z* 255.1217 ((*E*)-5-hydroxy-3-((8-hydroxy-2-methyleneoct-3-enoyl)oxy)-3,4-dihydro-2*H*-furan-1-ium, C_13_H_19_O_5_^+^), which was one of the diagnostic fragment ions of the predicted structure of ETO-carnitine shown in Fig. 2B. MS^3^ fragmentation ions arising from the *m/z* 255.1217 peak and their proposed theoretical fragments are shown in Fig. 2D and supplemental Fig. S1, which collectively identify and establish the chemical structure of ETO-carnitine.

### LC-HRAM MS^n^ analyses distinguish ETO-carnitine from hydroxy-C18:1 and hydroxy-C18:0 carnitines

**Fig. 3:**
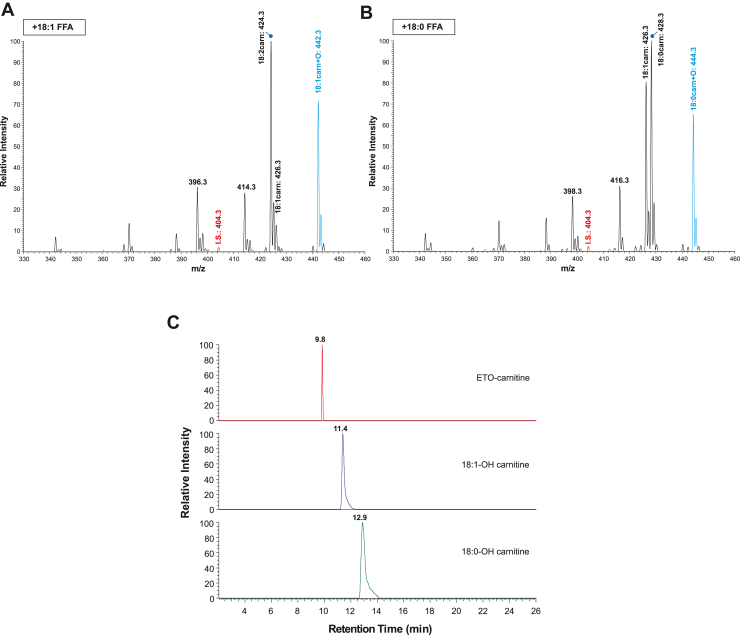
Mass spectrometric comparisons of etomoxir-carnitine to hydroxy-oleoylcarnitine and hydroxy-stearoylcarnitine. Isolated heart mitochondria were incubated with either 5 μM oleic acid or 5 μM stearic acid in a mitochondria assay buffer and fatty acylcarnitine products generated from mitochondrial fatty acid β-oxidation were extracted as described in “Materials and Methods.” The generation of hydroxy-oleoylcarnitine (A) and hydroxy-stearoylcarnitine (B) were monitored by TSQ MS spectrometry. Hydroxy-oleoylcarnitine and hydroxy-stearoylcarnitine (highlighted in blue) were identified at *m/z* values of 442 and 444, respectively, which were virtually identical to the *m/z* values of the two predominant etomoxir-carnitine isotopes shown in Figure 1. Palmitoyl-1,2,3,4-[^13^C_4_]-L-carnitine (*m/z* 404.3) was used as an internal standard (I.S. in red). C: Retention times for etomoxir-carnitine, hydroxy-oleoylcarnitine (C18:1-OH carnitine), and hydroxy-stearoylcarnitine (C18:0-OH carnitine) were determined and compared utilizing LC-MS. Extracted ion chromatograms for ETO-carnitine, C18:1-OH carnitine, and C18:0-OH carnitine with a 5 ppm mass window are shown.

**Fig. 4:**
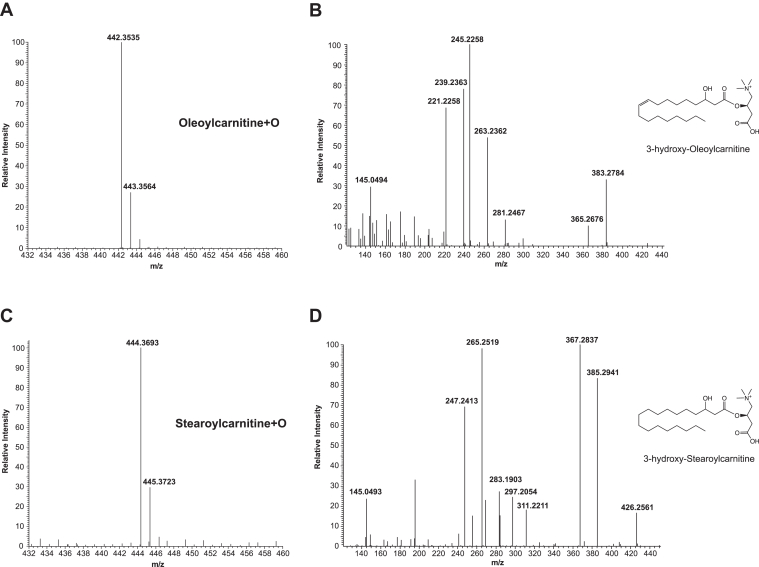
Accurate mass MS^2^ analysis distinguishes hydroxy-C18 fatty acylcarnitines from etomoxir-carnitine isotopes. Isolated heart mitochondria were incubated with either stearic acid (5 μM) or oleic acid (5 μM) in mitochondria assay buffer and fatty acylcarnitines were extracted as described in “Materials and Methods.” 3-hydroxy-oleoylcarnitine (C18:1-OH carnitine, MS^1^*m/z* 442.3535 (A)) and 3-hydroxy-stearoylcarnitine (C18:0-OH carnitine, MS^1^*m/z* 444.3693 (C)) were separated by LC-HRAM MS and their accurate masses determined. To distinguish these two β-oxidation products of oleic acid and stearic acid from the etomoxir-carnitine isotopes of similar nominal mass, fragmentation patterns for these products were determined by LC-HRAM MS^2^ analysis. B: MS^2^ spectrum of C18:0-OH carnitine identified as 3-hydroxy-oleoylcarnitine. D: MS^2^ spectrum of C18:1-OH carnitine identified as 3-hydroxy-stearoylcarnitine. The locations of the hydroxylated carbons were determined by the molecular ion fragmentation profiles shown in supplemental data.

Since the molecular masses of the two Cl isotopes of ETO-carnitine are similar to naturally occurring 3-hydroxy-C18:1 carnitine and 3-hydroxy-C18:0 carnitine, we examined the retention times and the accurate masses of hydroxy-C18 acylcarnitines. To generate hydroxy-C18:1 and hydroxy-C18:0 acylcarnitines, isolated mouse heart mitochondria were incubated with either oleic acid or stearic acid in the absence of ETO (Fig. 3). Mitochondrial fatty acid β-oxidation produced multiple downstream fatty acylcarnitines including hydroxy-C18:1 (*m/z* 442.3) and hydroxy-C18:0 acylcarnitines (*m/z* 444.3), which were generated from oleic acid and stearic acid, respectively. The LC-MS retention times of these acylcarnitines were clearly distinguishable from the two Cl isotopes of ETO-carnitine. The accurate masses of the hydroxy-C18 acylcarnitines observed by HRAM mass spectrometry were *m/z* 442.3535 (Δm/m = 1.81 ppm) and *m/z* 444.3693 (Δm/m = 2.03 ppm) for hydroxy-C18:1 and hydroxy-C18:0 acylcarnitines, respectively, which were distinct from the [^35^Cl]- and [^37^Cl]-ETO-carnitine isotopes. Finally, LC-MS/MS analyses revealed that the resultant fragmentation patterns of the hydroxy-C18 acylcarnitines identified the location of the hydroxy group and clearly indicated distinct differences in comparison to the MS^2^ profile of ETO-carnitine (Fig. 4 and supplemental Fig. S2).

Collectively, the two predominant peaks present in mitochondria treated with ETO were conclusively identified as the ^35^Cl and ^37^Cl isotopes of ETO-carnitine by LC-HRAM MS distinguishing them from endogenous hydroxy-C18 fatty acylcarnitines of identical nominal masses but with distinct and resolvable high mass accuracy *m/z* values, fragmentation patterns, and differential chromatographic behavior by UHPLC.

### ETO-carnitine possesses a reactive oxirane ring

**Fig. 5:**
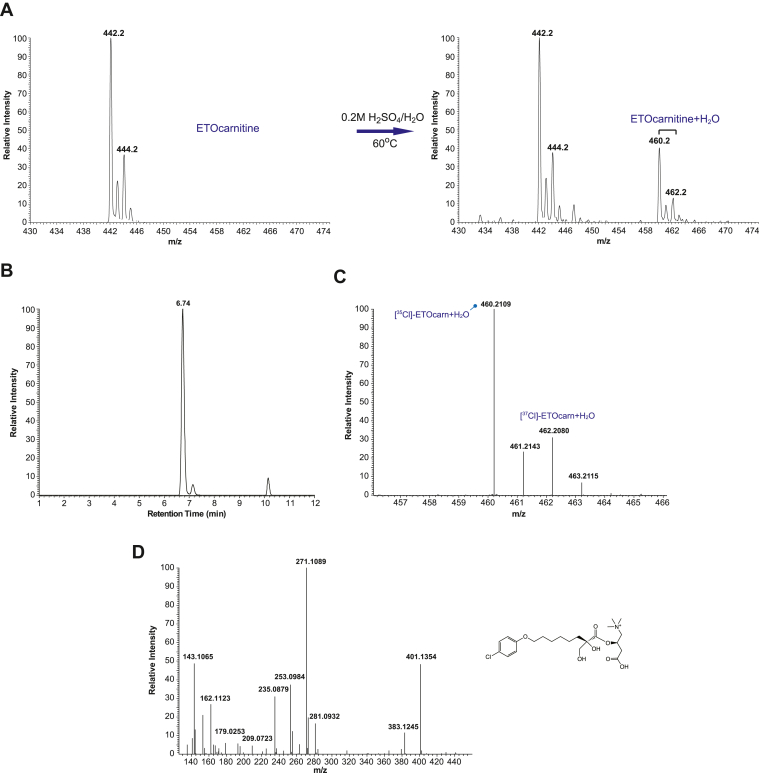
Identification of the intact oxirane ring in etomoxir-carnitine. The presence of the reactive oxirane ring in the isolated etomoxir-carnitine was confirmed by an acid-catalyzed hydrolytic reaction. Etomoxir-carnitine was prepared utilizing mouse heart mitochondria incubated with etomoxir and purified by HPLC. The purified etomoxir-carnitine (A, left panel) was reconstituted in a solution of 0.2 M H_2_SO_4_ and heated at 60°C for 2 h. The resultant products were immediately measured by TSQ ESI-MS spectrometry. The generation of the water-adduct of ETO-carnitine (dihydroxy etomoxir-carnitine) was confirmed by the presence of peaks at *m/z* 460 and *m/z* 462 representative of the two Cl isotopes of [etomoxir-carnitine + H_2_O]^+^ (A, right panel). B: LC retention time of ring-opened ETO-carnitine for analysis by LC-MS. Intact ETO-carnitine was incubated in an acidic solution with heating, and the resultant products were analyzed by LC-MS and LC-MS^2^ as described in “Materials and Methods”. The peak for [M + H_2_O]^+^ eluted at 6.74 min, which is significantly shorter than the retention time of ETO-carnitine, M^+^(9.84 min, Fig. 2A). C: Accurate mass of ring-opened ETO-carnitine was determined by Orbitrap MS analysis which identified the two Cl isotopes of [M + H_2_O]^+^ (i.e., [^35^Cl]-ETOcarn + H_2_O and [^37^Cl]-ETOcarn + H_2_O). D: A representative spectrum for the fragments of *m/z* 460.2109 by MS/MS tandem mass spectrometric analysis is shown with the predicted chemical structure of ring-opened ETO-carnitine (right).

ETO possesses a reactive oxirane group that is likely critical for the covalent inhibition of CPT1. Prior work has suggested a putative mechanism by which ETO-CoA, an activated (thioesterified) form of ETO, covalently modifies a nucleophilic serine (Ser723) within the CPT1 active site resulting in irreversible inhibition of CPT1 (12). However, it has not been unambiguously proven whether ETO-CoA is a product of thioester formation through the ETO carboxylic acid by ACSL or through reaction of CoASH with the epoxide of ETO by nucleophilic-induced opening of the oxirane ring (24). To determine if ETO-carnitine still retained the highly reactive oxirane ring, we treated HPLC-purified ETO-carnitine with an acidic aqueous solution at 60°C for 2 h as described in “Materials and Methods”. The resultant product was immediately measured by TSQ ESI-MS. New carnitine-containing products following heating in the presence of acid were detected as the two Cl isotopes of the water adduct of ETO-carnitine [ETO-carnitine + H_2_O] (Fig. 5A). The identities of these reaction products were then further analyzed by LC-HRAM MS. The retention time for the water-adduct of ETO-carnitine (dihydroxy ETO-carnitine) was 6.74 min, which was markedly shorter than the retention times of either intact ETO-carnitine, hydroxy-C18:1carnitine, or hydroxy-C18:0 carnitine (Figs. 3C and 5B). The masses of the water-adducts of the two Cl isotopes of ETO-carnitine matched their expected theoretical masses within 5 ppm mass error (i.e., observed *m/z* of 460.2109 (Δm/m = 2.61 ppm) and 462.2080 (Δm/m = 2.81 ppm) versus calculated *m/z* of 460.2097 and 462.2067, respectively) (Fig. 5C). Furthermore, MS^2^ fragmentation patterns distinguished the water-adduct product of ETO-carnitine from nonhydrolyzed ETO-carnitine and hydroxy-C18 acylcarnitines identifying it as (*R*)-3-carboxy-2-(((*R*)-8-(4-chlorophenoxy)-2-hydroxy-2-(hydroxymethyl)octanoyl)oxy)-*N*,N,*N*-trimethylpropan-1-aminium, C_22_H_35_ClNO_7_^+^ (Fig. 5D and supplemental Fig. S3).

### ETO-carnitine production is dependent on ACSL and CPT1

**Fig. 6:**
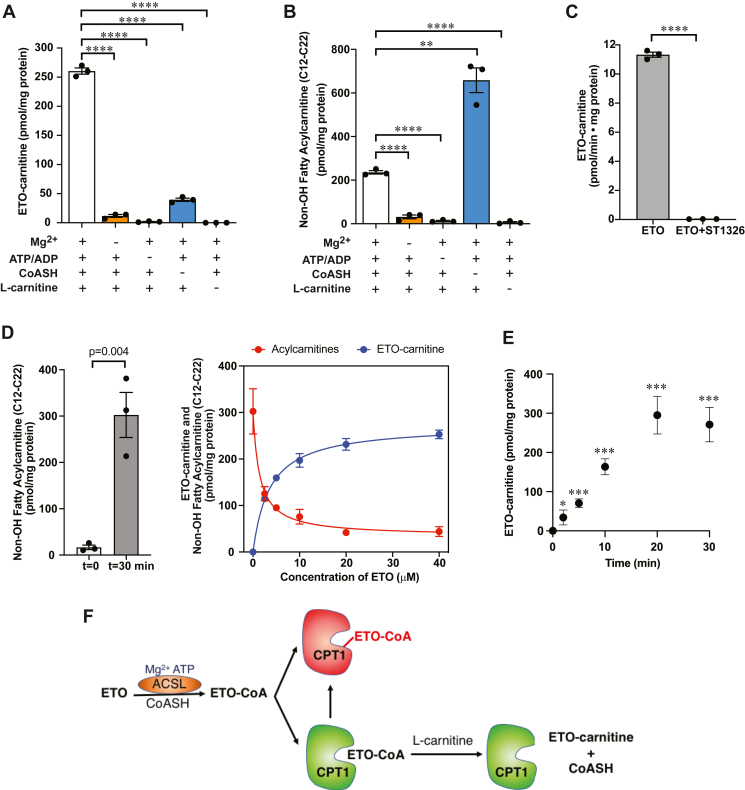
Production of Etomoxir-carnitine in intact mitochondria is dependent upon acyl-CoA synthetase and CPT1 activities. A: Intact heart mitochondria were isolated and incubated in isotonic buffer containing 5 mM succinate, 40 μM etomoxir, and 2 μM rotenone in the presence or absence of 5 mM MgCl_2_, 0.5 mM ATP/1.25 mM ADP, 0.5 mM reduced coenzyme A (CoASH), or 0.5 mM L-carnitine as indicated for etomoxir-carnitine (ETO-carnitine) generation determined as described in “Materials and Methods”. B: Mitochondrial fatty acylcarnitines were generated from isolated mitochondria which were incubated in the isotonic mitochondria buffer in the presence or absence of 5 mM MgCl_2_, 0.5 mM ATP/1.25 mM ADP, 0.5 mM CoASH, and/or 0.5 mM L-carnitine without ETO as indicated. The quantities of mitochondrial fatty acylcarnitines were determined using ESI-MS with palmitoyl-1,2,3,4-[^13^C_4_]-L-carnitine as an internal standard. C: ST1326, a reversible CPT1 inhibitor, was utilized to determine the CPT1-dependency of etomoxir-carnitine production. Intact mitochondria (120 μg protein) isolated from murine heart tissue were incubated with 40 μM etomoxir in the presence or absence of 25 μM ST1326 for 15 min. The amount of etomoxir-carnitine generated was determined by LC-MS utilizing palmitoyl-1,2,3,4-[^13^C_4_]-L-carnitine as an internal standard as described in “Materials and Methods”. Significance of comparisons of the bracketed data sets are as indicated (∗∗*P* < 0.01 and ∗∗∗∗*P* < 0.0001). D: Inhibition of mitochondrial acylcarnitine production and the generation of etomoxir-carnitine were measured using increasing concentrations of etomoxir. Generation of total measured nonoxidized fatty acylcarnitines during incubation for 30 min is shown compared with initial amounts at time = 0 (left panel). The decrease in fatty acylcarnitines (red line) at 2.5, 5, 10, 20, 40 μM etomoxir was monitored by TSQ ESI mass spectrometry and the generation of etomoxir-carnitine was measured by LC-MS (blue line). (N = 3, mean ± S.E.M). E: Time course of ETO-carnitine production in intact heart mitochondria incubated with 10 μM ETO for the indicated times. The amount of ETO-carnitine produced at each time point was determined by LC-MS and normalized to mg of total mitochondrial protein. (N = 3, mean ± S.E.M). Significance of results when compared with controls at t = 0 min are as indicated (∗*P* < 0.05 and ∗∗∗*P* < 0.001). F: Etomoxir-carnitine is hypothesized to be generated via CPT1 following ACSL-catalyzed formation of etomoxir-CoA (lower pathway with CPT1 in green). Mitochondrial production of etomoxir-carnitine is terminated at the point of complete inhibition of CPT1 (red) by etomoxir-CoA.

In order to establish the mitochondrial enzymes necessary for ETO-carnitine synthesis, we examined the various substrates and cofactors that were required for ETO-carnitine production. Mouse heart mitochondria were incubated with ETO in the absence or presence of MgCl_2_, ATP/ADP, coenzyme A (CoASH), or L-carnitine. Notably, mitochondria required each of these components to generate ETO-carnitine from ETO. These results strongly suggest that long-chain acyl-CoA synthetase (which requires Mg^2+^/ATP and coenzyme A) activity catalyzes the generation of the ETO-CoA thioester from ETO prior to generation of ETO-carnitine (Fig. 6A). Interestingly, fatty acylcarnitine production from endogenous fatty acids in mitochondria (i.e., existing as free fatty acids or those released from membrane phospholipids by activated mitochondrial phospholipases A_1_/A_2_) did not require exogenous coenzyme A suggesting that fatty acids present in the mitochondrial intermembrane space are likely processed for fatty acid β-oxidation utilizing endogenous coenzyme A (CoASH) stored in the mitochondrial matrix, CoASH present in the intermembrane space, or CoASH released from endogenous CoA-containing metabolites. In contrast, mitochondrial synthesis of ETO-carnitine required exogenous coenzyme A for the conversion of exogenous ETO to its carnitine derivative presumably due to the inability of ETO to traverse mitochondrial membranes (Fig. 6B). Utilization of an independent reversible CPT1 inhibitor, ST1326 (25), completely blocked the production of ETO-carnitine, indicating that CPT1 catalyzes ETO-carnitine synthesis (Fig. 6C). Taken together, these results indicate that CPT1 catalyzes ETO-carnitine formation.

Next, we compared the dose-response profile for the production of ETO-carnitine versus endogenous fatty acylcarnitines in mitochondria treated with increasing concentrations of ETO. The IC_50_ for ETO-inhibited CPT1 has been reported to vary from subnanomolar to tens of micromolar concentration in a cell-type and tissue-specific manner that also is dependent on the assay methods utilized for the quantitation of CPT1 activity (8, 26). In the current work, we determined CPT1 activity from mouse heart mitochondria by measuring the production of nonoxidized fatty acylcarnitines (C12-C22) by mass spectrometry with a precursor ion scan using a known carnitine fragment at *m/z* 85.0. Quantitation of ETO-carnitine in purified mitochondria treated with ETO was performed in parallel by LC-MS. As expected, intact mouse heart mitochondria accumulated long-chain fatty acylcarnitines (C12-C22) utilizing endogenous mitochondrial fatty acids during 30 min incubation in the absence of ETO (Fig. 6D, left panel). The presence of ETO dramatically inhibited the production of endogenous fatty acylcarnitines in a concentration-dependent manner (IC_50_ ∼ 1.4 μM). In contrast, ETO-carnitine production was correspondingly increased mirroring the decrease in acylcarnitines mediated by ETO in intact mitochondria (Fig. 6D, right panel). At concentrations >20 μM, ETO and its metabolic derivatives (i.e., ETO-CoA and ETO-carnitine) nearly completely inhibited fatty acylcarnitine production. A time-course analysis of ETO-carnitine production in isolated mouse heart mitochondria utilizing 10 μM ETO revealed that ETO-carnitine increased linearly for 20 min before reaching a plateau (Fig. 6E). Inhibition of fatty acylcarnitine synthesis by ETO likely occurs from a combination of the competition of ETO with endogenous fatty acids in generating fatty acyl-CoAs (along with ETO-CoA) by ACSL as well as covalent addition of the ETO oxirane ring to nucleophilic residues within the CPT1 active site. This results in enzymatic ETO-carnitine production until CPT1 is irreversibly and nearly completely inhibited by the activated electrophilic form(s) of ETO (*e.g*., ETO-CoA). Collectively, these results strongly support the ACSL/CPT1-catalyzed enzymatic biosynthesis of ETO-carnitine in mitochondria (Fig. 6F).

### ETO-carnitine potently inhibits calcium-independent phospholipases A_2_

Previous X-ray crystallographic studies suggested that the structure of ETO-modified CPT1 contains a ring-opened oxirane ring covalently adducted to a serine residue (Ser723) near the active site of human CPT1B (12). Accordingly, we postulated that the active site nucleophilic residues of serine esterases could similarly attack the oxirane group of ETO. Furthermore, ETO-carnitine, in contrast to ETO, is able to cross the mitochondrial outer membrane through the carnitine transport system.

**Fig. 7:**
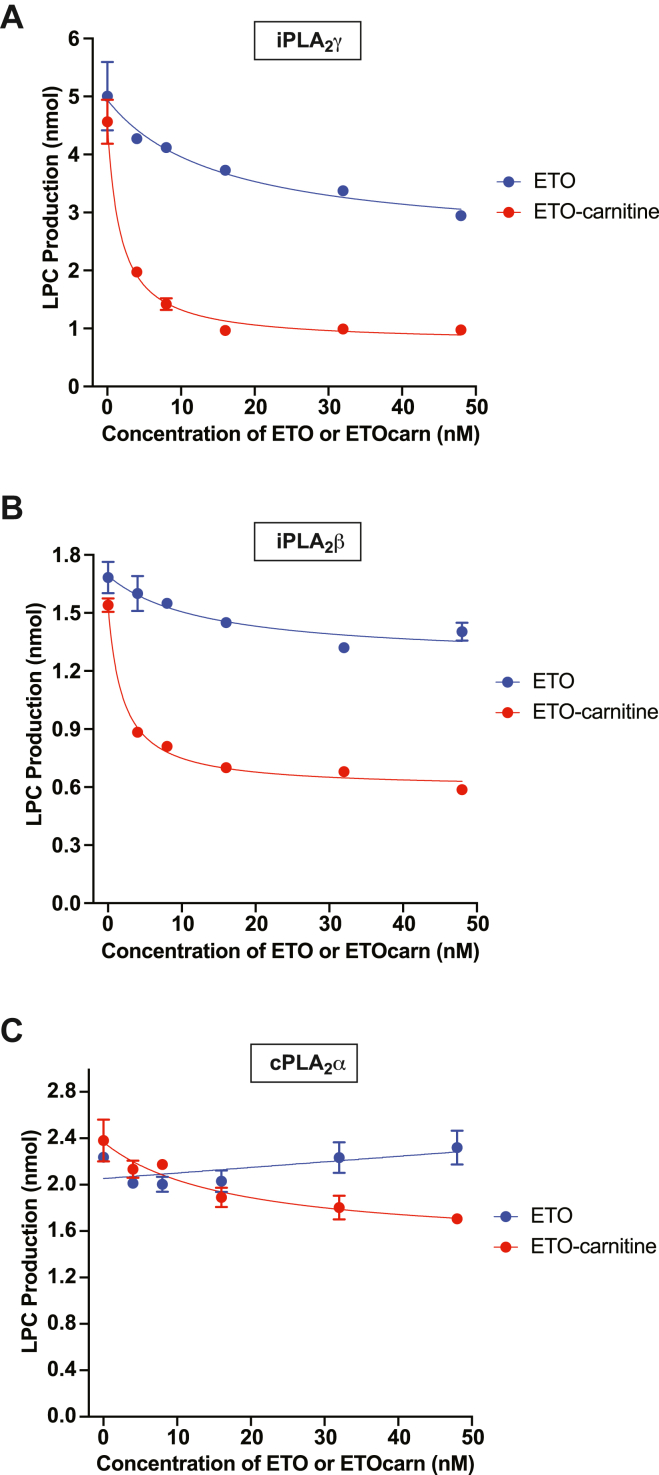
Etomoxir-carnitine potently inhibits calcium-independent phospholipase A_2_γ activity. Purified recombinant peroxisomal iPLA_2_γ (63 kDa, 0.5 μg) (A) or recombinant iPLA_2_β (80 kDa, 0.64 μg) (B) was incubated with 50 μM 1-palmitoyl-2-arachidonoyl-*sn*-glycero-3-phosphocholine (PAPC)/12 μM 1,3-diolein in reaction buffer containing 10 mM Hepes (pH 7.4), 10% glycerol, 50 mM KCl, 1 mM DTT for 10 min at the indicated concentrations (0, 4, 8, 16, 32, 48 nM) of either ETO (blue circles) or ETO-carnitine (red circles). Phospholipase A_2_ activity was quantitated by measuring total lysoPC (LPC) production (i.e., palmitoyl-LPC and arachidonoyl-LPC). C: Purified recombinant cPLA_2_α (85 kDa, 0.68 μg) was incubated with 50 μM PAPC/12 μM 1,3-diolein in reaction buffer containing 10 mM Hepes (pH 7.4), 10% glycerol, 50 mM KCl, and 0.5 mM CaCl_2_ for 10 min at the indicated concentrations (0, 4, 8, 16, 32, 48 nM) of either ETO (blue circles) or ETO-carnitine (red circles). The activity of cPLA_2_α was determined by measuring total LPC production (i.e., palmitoyl-LPC and arachidonoyl-LPC). Data are presented as mean ± S.E.M (N = 3).

Thus, we hypothesized that mitochondrial serine hydrolases such as the iPLA_2_s were potential candidates for nucleophilic addition reactions to ETO-carnitine resulting in phospholipase inhibition. We therefore examined the effect of ETO-carnitine on purified calcium-independent phospholipases A_2_ (i.e., iPLA_2_γ and iPLA_2_β) as well as cPLA_2_α (Fig. 7). Purified recombinant iPLA_2_γ, iPLA_2_β, and cPLA_2_α were generated in our laboratory (13, 14, 15) and incubated with various concentrations of either ETO or ETO-carnitine. Interestingly, iPLA_2_γ was potently inhibited by low nanomolar concentrations of ETO-carnitine with an apparent IC_50_ ∼ 1.75 nM, while nonesterified ETO only modestly inhibited iPLA_2_γ in the nanomolar concentration range (Fig. 7A). Low nanomolar concentrations of ETO-carnitine also inhibited iPLA_2_β by ∼60% (Fig. 7B). In stark contrast, ETO essentially had a diminutive effect on cPLA_2_α activity in the concentration range examined, although ETO-carnitine modestly but significantly inhibited cPLA_2_α at > 40 nM concentration (Fig. 7C).

### Mitochondrial respiration with palmitoylcarnitine is inhibited by ETO-carnitine in a CPT1-independent manner

**Fig. 8:**
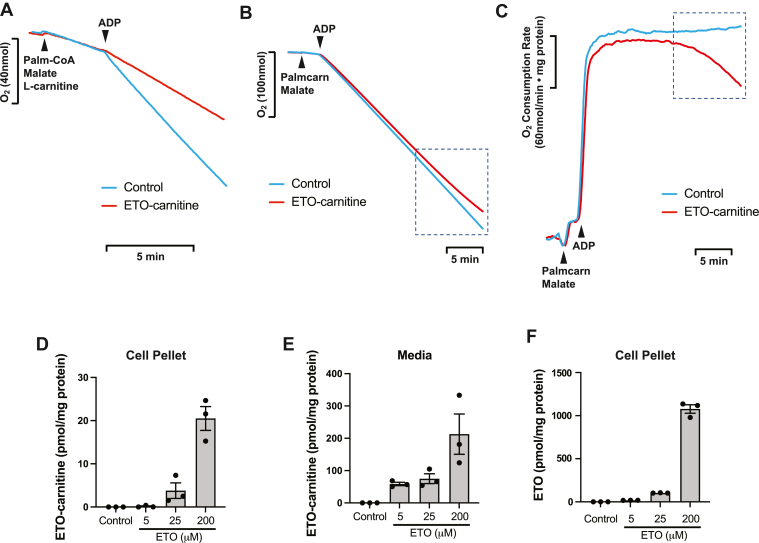
Etomoxir-carnitine inhibits mitochondrial respiration independent of CPT1 utilizing palmitoylcarnitine as the substrate. Intact mitochondria were isolated from mouse heart and reconstituted in mitochondrial respiration buffer (MiR05) in an OROBOROS chamber (2 ml) in the absence (DMSO vehicle, Control) or presence of 0.5 μM etomoxir-carnitine (ETO-carnitine) as described in “Materials and Methods”. After 10 min pre-incubation with ETO-carnitine, mitochondria were exposed to the substrates, palmitoyl-CoA (Palm-CoA)/malate/L-carnitine (A) or palmitoylcarnitine (Palmcarn)/malate (B), to determine state 2 respiration followed by the addition of ADP for ADP-activated state 3 respiration at the indicated times (arrows). The decrease in the concentration of O_2_ in the chamber (A and B) and the O_2_ consumption rate (C) were recorded. ETO-carnitine markedly inhibited CPT1-dependent mitochondrial respiration with palmitoyl-CoA (A). ETO-carnitine induced delayed but significant inhibition of mitochondrial respiration with palmitoylcarnitine (dotted box) (B, C). Representative tracings are shown from three independent measurements. Production of ETO-carnitine by HepG2 cells as indicated by the accumulation of ETO-carnitine in the cell pellet (D) and in the media (E) following incubation of cells in the presence of 0, 5, 25, or 200 μM ETO was determined by LC-MS and normalized to the amount of protein present in the cell pellet as described in “Materials and Methods”. F: The accumulation of intracellular ETO (nonmetabolized) in HepG2 cells was determined by LC-MS at the indicated concentrations of ETO and normalized to the amount of protein present in the cell pellet. Data in panels D–F are presented as mean ± S.E.M (N = 3) for each condition.

Next, we investigated whether ETO-carnitine impacted mitochondrial respiration utilizing palmitoylcarnitine as substrate in the absence or presence of ETO-carnitine (Fig. 8). Palmitoylcarnitine, the ACSL-CPT1 product of palmitate, does not require CPT1 for transport into mitochondria and serves as a direct substrate for mitochondrial FAO and respiration for energy production (8, 27). Since ETO is known as a highly selective irreversible CPT1 inhibitor that potently inhibits fatty acid- or fatty acyl-CoA-facilitated respiration, it would be predicted to not inhibit palmitoylcarnitine-mediated respiration in isolated mitochondria (8, 27). However, treatment of heart mitochondria with ETO-carnitine not only potently inhibited CPT1-dependent state 3 respiration with palmitoyl-CoA as substrate, but also progressively inhibited mitochondrial respiration with palmitoylcarnitine as substrate which accelerated over time (Fig. 8A–C). Considering that palmitoylcarnitine does not use CPT1 for mitochondrial respiration, this process may indicate that the accumulation of ETO-carnitine within a particular mitochondrial microenvironment, enzyme complex, or other nucleophilic sites within the mitochondrial matrix is able to inhibit mitochondrial oxidative phosphorylation in a CPT1-independent manner. Furthermore, we demonstrated that HepG2 cells treated with ETO at various concentrations produced ETO-carnitine in a concentration-dependent manner and released it into extracellular space (Fig. 8D–F). Collectively, ETO-carnitine derived from ETO was able to inhibit mitochondrial respiration by a mechanism independent of CPT1 which may occur through inhibition of mitochondrial serine esterase(s) (such as iPLA_2_γ) or alteration of the conformational state of active site further suggesting that this novel pharmaco-metabolite impedes critical enzymes in mitochondrial fatty acid uptake/release, FAO processing, and/or ETC bioenergetics.

## Discussion

Etomoxir has been described as a selective inhibitor of CPT1 and thus has been widely used to examine the functional roles of fatty acid oxidation in multiple disease states including cardiac ischemia, heart failure, diabetes, and obesity. Specifically, because long-chain fatty acids are only slowly permeable across the mitochondrial double membrane and their activated forms (i.e., acyl-CoA) are membrane-impermeable in the absence of CPT1, ETO has been extensively utilized to assess the role of mitochondrial fatty acid β-oxidation in numerous disease states.

The mechanism for the inhibition of CPT1 by ETO has been studied extensively (12), although our understanding of its downstream effects remains incomplete. Etomoxir present in the cytosol (or within cellular membranes) is first activated to ETO-CoA by long-chain ACSLs. Etomoxir-CoA is believed to directly interact with CPT1 since it can bind to the long chain acyl-CoA-binding site of CPT1 (28, 29, 30). It has been proposed that the highly reactive oxirane ring of ETO-CoA is next covalently adducted to a serine residue within the active site of CPT1 resulting in the irreversible inactivation of CPT1. Despite the apparent inhibitory potency of ETO for CPT1, multiple other studies have reported unanticipated off-target effects from the use of ETO that are associated with its use. For example, high concentrations of ETO have been demonstrated to reduce the proliferation rate of breast cancer cells independently of the rate of fatty acid oxidation (11). Moreover, ETO at >10 μM has been demonstrated to inhibit the mitochondrial adenine nucleotide transporter and ETC complex I resulting in oxidative stress (8, 11, 31). Concentrations of ETO in excess of 5 μM have been shown to induce acute production of ROS with evidence of severe oxidative stress in proliferating T cells and increase markers of oxidative/ER stress in human liver slices (9, 32). ETO has also been shown to have off-target effects on DGAT1 in primary mouse hepatocytes in addition to the inhibition of fatty acid β-oxidation resulting in inhibition of medium chain fatty acid triglyceride synthesis (33). In addition, ETO has been found to increase the expression of adipose differentiation-related protein in the liver tissue of treated rats (34). The toxicity of ETO (producing systemic side effects) is well-established thereby preventing it from being approved for future clinical trials (35, 36, 37).

Conventional assays of CPT1 activity to investigate the effects of ETO are typically performed by measuring cellular/mitochondrial oxygen consumption (respiration) or utilizing stable isotopes of fatty acids and/or their carnitine derivatives for analysis of the metabolic flux of mitochondrial fatty acid β-oxidation. These determinations of CPT1 activity depend heavily on the presumed specificity of ETO. However, like all pharmacologic agents, off-target effects cannot be ruled out. Previously, it was unknown whether the active form of ETO, ETO-CoA, was further processed as a CPT1 substrate in mitochondria (as fatty acylcarnitines). In this study, we hypothesized that ETO-CoA was further metabolized by CPT1 to ETO-carnitine due to the presence of a thioester linkage in ETO-CoA. Aliphatic CoA thioesters have a high affinity for CPT1, which normally facilitates the transfer of the aliphatic group of acyl-CoA to carnitine to form the corresponding aliphatic carnitines. Accordingly, we postulated that the domain of CPT1 responsible for thioester binding and transfer of carnitine can be utilized for the transfer of carnitine to ETO-CoA to form ETO-carnitine. We utilized high-resolution mass spectrometry to monitor CPT1-catalyzed generation of ETO-carnitine and CPT1 inhibition by ETO through measuring the final products of the CPT1 reaction (i.e., fatty acylcarnitines). Substrate/product profiles and the cofactor requirements for the production of ETO-carnitine demonstrated that ETO-CoA (generated by ACSL activity which thioesterifies ETO with CoASH (12, 23)) was either further metabolized by CPT1 into ETO-carnitine or adducted to CPT1 resulting in its inactivation. Moreover, the generation of ETO-carnitine by CPT1 on the cytosolic face of mitochondria suggested that it could inhibit not only untargeted mitochondrial enzymes present in the intermembrane space and in the inner membrane, but cytosolic enzymes as well. Furthermore, we demonstrated the generation, accumulation, and rapid release of ETO-carnitine as well as fatty acylcarnitines from HepG2 cells into the extracellular space. This result suggests more complex biochemical effects of ETO and its metabolites which can pass through cellular membranes by a number of mechanisms including transport systems, transmembrane flip-flop, or vesicle-mediated delivery of cargo for specific molecular processes. Collectively, the multiple pharmaco-metabolites of ETO likely have access to almost all cellular compartments including membrane domains, cytosolic metabolons, organelles, and transport/signaling vesicles. In essence, ETO acts as a latent pharmacologic substance that is ultimately activated by thioesterification to CoA and/or by subsequent esterification to carnitine prior to diffusion from the membrane compartments from which they are generated.

During the course of our studies with ETO, we identified moieties initially believed to be hydroxy-C18:0 and hydroxy-C18:1 acylcarnitines having nominal masses, *m/z* 444 and *m/z* 442, respectively. Subsequent investigation of these moieties with high-resolution approaches demonstrated that they were in fact two distinct Cl isomers of ETO-carnitine (^35^Cl and ^37^Cl) that were present only after treatment of mitochondria with ETO. Multiple lines of evidence including elemental analysis, mass spectrometric identification of ETO-carnitine and demonstration of the intact oxirane ring in ETO-carnitine substantiated these results.

Specific pharmacologic inhibitors can often provide critical insight into the importance of a target enzyme on the biologic process being studied. However, the utility of pharmacologic inhibitors is dependent on their specific and high affinity interactions with the target enzyme. Typically, this requires careful titration of the inhibitor concentration so that the observed effects reflect solely the inhibition of the targeted enzyme. However, in some cases, off target-effects can be present at considerably lower inhibitor concentrations than that needed for inhibition of the targeted enzyme.

Although the identification of a new pharmacologic product is in itself an important finding, we additionally postulated that the intact oxirane ring of ETO-carnitine could be subject to nucleophilic attack by enzymes other than CPT1. One of the most intriguing findings of the current study is that low nanomolar concentrations of ETO-carnitine can potently inhibit iPLA_2_β or iPLA_2_γ. One contribution to this unanticipated finding is the structural similarity of ETO-carnitine to the natural phospholipid substrates of iPLA_2_s, such as phosphatidylcholine (PC), which likely share similar stereoelectronic relationships at the membrane interface.. Specifically, these structural components include: 1) a positively-charged quaternary amine; 2) a region of negative charge density at the membrane interface (provided by the phosphate in PC or by the carboxylate in ETO-carnitine); and 3) a hydrophobic acyl chain. Critically, the quaternary amine and carboxylate are in close spatial proximity which likely facilitates increased binding and dwell time in the iPLA_2_ active site. Tight non-covalent interactions with the iPLA_2_ substrate binding site may also be facilitated through the hydrophobic acyl group of ETO-carnitine. Notably, iPLA_2_β or iPLA_2_γ each have an active site serine which normally participates in the release of the fatty acid product. However, in the case of ETO-carnitine, the active site serine (or surrounding nucleophilic residues) may attack the oxirane ring of the ETO moiety thereby resulting in elimination of phospholipase activity. The use of oxiranes for inhibition of serine lipases has previously been utilized in synthetic oxirane-containing lipids (38). Such inhibition of off-target enzymes is typically traversed by lowering the concentration of the inhibitor. However, in the present case, it seems clear that off-target effects cannot be overcome by simply lowering concentrations of the inhibitor since the IC_50_ of ETO-carnitine for off-target effects (e.g., inhibition of iPLA_2_β and iPLA_2_γ) occur orders of magnitude less than the IC_50_ of ETO for apparent inhibition of CPT1 activity through measurement of mitochondrial fatty acylcarnitine production. Thus, when fatty acid β-oxidation is apparently inhibited by ETO, the resultant metabolic and signaling effects are not due exclusively to the inhibition of CPT1, but in addition likely reflect alterations in cell signaling and mitochondrial bioenergetics at concentrations lower than the purported target site resulting in off-target inhibition of potentially a panoply of enzymes. Previous work has identified the ability of high concentrations of ETO (50–100 μM) to inhibit lipolysis and triacylglycerol secretion in adipocytes, possibly indicating the targeting of ATGL and other lipases by ETO as well as other off-target effects (39). The significance of these results is apparent in studies using high concentrations of ETO to inhibit fatty acid oxidation (through CPT1) where off-target effects occur and likely result from, at least in part, ETO-carnitine which is highly susceptible to nucleophilic attack of the oxirane moiety (either enzyme-catalyzed or non-enzymic). We specifically note that these represent only a few of the off-target effects of ETO which likely effects multiple enzymes and metabolic pathways. Etomoxir has been extensively used to interrogate the importance of lipid metabolism in diverse biological systems which is thought to be due largely through CPT1 loss of function and inhibition of FAO. The results of the present study identify the nature of mixed inhibition of various enzymes and functions due to off-target effects of ETO.

Importantly, we found that ETO-carnitine potently inhibited mitochondrial respiration with palmitoylcarnitine as substrate. Palmitoylcarnitine is a product of CPT1 catalysis and can be readily transported into the mitochondrial matrix for FAO through carnitine-acylcarnitine translocase (CACT) and CPT2 activities acting sequentially in a process which would be predicted to be CPT1-independent (8, 27). The results from this study open the possibility that ETO-carnitine, which is more readily transported than ETO through membrane translocases like CACT, can affect multiple steps of FAO utilizing acylcarnitine/acyl-CoA as substrate, and/or the mitochondrial respiration machinery directly. In support of these results, previous studies by our group and others have reported CPT1-independent disruption of mitochondrial respiration by ETO implicating its ability to inhibit adenine nucleotide transferase and/or electron transport chain complexes (11, 31, 40).

Collectively, this study identifies a mitochondrial metabolite of ETO, ETO-carnitine, utilizing multiple independent approaches to substantiate its molecular structure and definitively distinguish it from naturally occurring fatty acid β-oxidation products of nominal mass. High mass accuracy MS was utilized to distinguish ETO-carnitine from hydroxy-C18:1carnitine and hydroxy-C18:0 carnitine. This result emphasizes the necessity of high mass accuracy MS analyses when identifying/quantifying fatty acylcarnitines in biologic systems interrogated with ETO. Furthermore, ETO-carnitine possesses different inhibitory potencies in comparison to unconjugated ETO toward various enzymic targets and exhibits altered membrane transport properties which likely results in direct off-target effects in multiple cellular compartments as well as in mitochondria.

## Data availability

All data described in this study are contained in the manuscript.

## Supplemental data

This article contains supplemental data.

## Conflict of interests

The authors declare that they have no conflicts of interest with the contents of this article.
