# Supplementary material for: Etomoxir-carnitine, a novel pharmaco-metabolite of etomoxir, inhibits phospholipases A2 and mitochondrial respiration
Source: J Lipid Res. 2024 Jul 31;65(9):100611. doi: 10.1016/j.jlr.2024.100611 (PMC11402452; doi:10.1016/j.jlr.2024.100611)
Supplement: Supplementary data [file mmc1.pdf]

Figure S1.

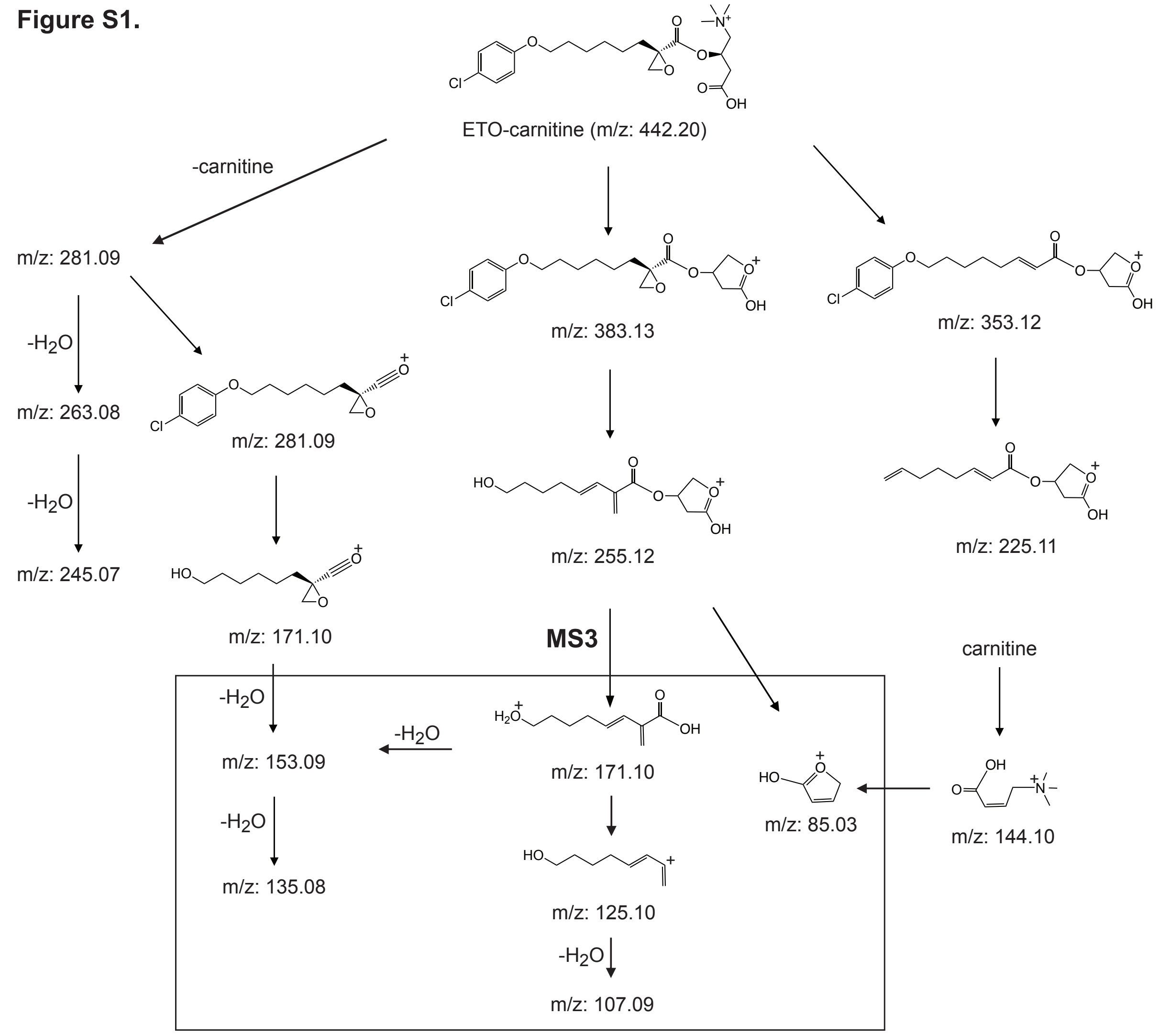

Supplemental Figure S1. Proposed MS^2 fragmentation for etomoxir-carnitine, followed by MS^3 fragmentation for the MS^2 fragment ion at m/z=255.12.

Figure S2.

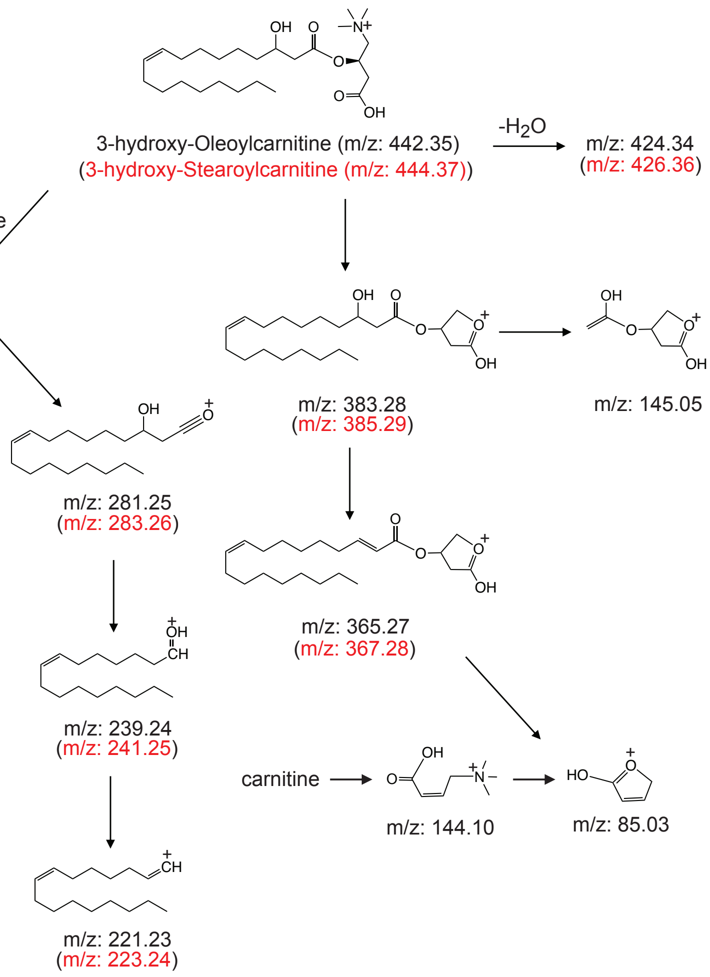

Supplemental Figure S2. Proposed MS^2 fragmentation for 3-hydroxy-Oleoylcarnitine and 3-hydroxy-Stearoylcarnitine.

Figure S3.

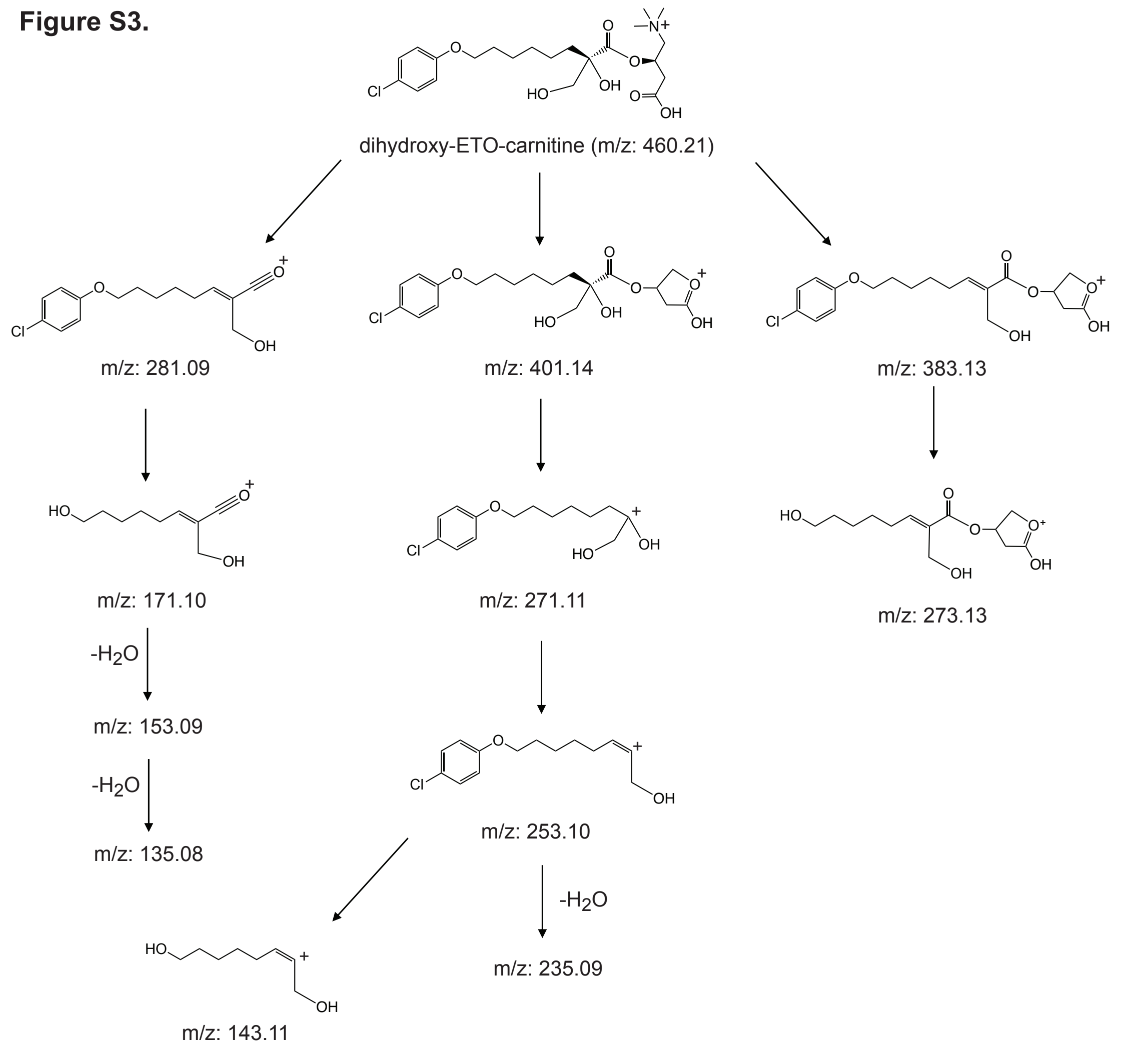

Supplemental Figure S3. Proposed MS^2 fragmentation for the water-adduct of etomoxir-carnitine (dihydroxy-ETO-carnitine).
